# Probiotics and Their Potential Preventive and Therapeutic Role for Cancer, High Serum Cholesterol, and Allergic and HIV Diseases

**DOI:** 10.1155/2018/3428437

**Published:** 2018-08-29

**Authors:** Yusuf Nazir, Syed Ammar Hussain, Aidil Abdul Hamid, Yuanda Song

**Affiliations:** ^1^Colin Ratledge Center for Microbial Lipids, School of Agriculture Engineering and Food Science, Shandong University of Technology, Zibo 255049, China; ^2^School of Biosciences and Biotechnology, Faculty of Science and Technology, Universiti Kebangsaan Malaysia, Malaysia

## Abstract

The potential health benefits of probiotics have long been elucidated since Metchnikoff and his coworkers postulated the association of probiotic consumption on human's health and longevity. Since then, many scientific findings and research have further established the correlation of probiotic and gut-associated diseases such as irritable bowel disease and chronic and antibiotic-associated diarrhea. However, the beneficial impact of probiotic is not limited to the gut-associated diseases alone, but also in different acute and chronic infectious diseases. This is due to the fact that probiotics are able to modify the intestinal microbial ecosystem, enhance the gut barrier function, provide competitive adherence to the mucosa and epithelium, produce antimicrobial substances, and modulate the immune activity by enhancing the innate and adaptive immune response. Nevertheless, the current literature with respect to the association of probiotic and cancer, high serum cholesterol, and allergic and HIV diseases are still scarce and controversial. Therefore, in the present work, we reviewed the potential preventive and therapeutic role of probiotics for cancer, high serum cholesterol, and allergic and HIV diseases as well as providing its possible mechanism of actions.

## 1. Introduction

The association of live-microbial feed with well-being has a long history which dated back to thousands of years ago [1]. However, the use of word “probiotic” was first introduced in 1974 by Parker who defined it as organisms and substances that have a beneficial effect on the host animal by contributing to its intestinal microbial balance and since then, the definition of probiotic has been improved several times [2]. The first documented study on probiotic was reported by Metchnikoff and his coworkers who discussed his view on the lower gut flora and the beneficial effects of fermented milk on human's health and longevity. Therefore, he was awarded the Nobel Prize in Medicine in 1908 for his cellular (phagocytic) theory of immunity and has inspired generations of scientists and food product developers with his proposal to transform the “toxic” flora of the large intestine into a host-friendly colony of* Bacillus bulgaricus *[3]. Since then, extensive studies on the beneficial effect of probiotics on human have been conducted and its relation in preventing and treating gut-related diseases such as infectious and antibiotic-associated diarrhea, irritable bowel diseases, lactose intolerance, indigestion, and stomach bloating have been established [4]. Due to the significant role of probiotic in enhancing the gut health and overall human well-being, the demand for probiotic-based nutriment has increased tremendously. In 2007, the global market for probiotic ingredients, supplements, and foods worth was $14.9 billion and it augmented up to US$16 billion in 2008. Furthermore, the probiotics market is anticipated to expand from $37.7 in 2016 to $71.9 billion by 2025, at a CAGR of 7.49% [5, 6].

In addition to improving the gut health, probiotics have also been documented to exert other health-promoting effects including chronic diseases such as cancer [7–9], high serum cholesterol [10], and allergy [11] and slowing the disease progression and symptoms of the HIV-infected individual such as bacterial translocations as well as vulvovaginal candidiasis in women [12]. Preventive and therapeutic role of probiotic on cancer has been established via several mechanisms including modulation of gut microbiota, enhancement of gut barrier functions, degradation of potential carcinogens and enhancement of immune system [7]. For instance, a study by Ma et al. [8] found that probiotic* Bacillus polyfermenticus* exerts an anticancer effect on human colon cancer cells stimulating IgG production and modulates the number of CD4þ, CD8þ, or NK cells. In another study involving 54 women found that a daily probiotic consumption for 6 months enhanced the clearance of human papillomavirus (HPV) which is known to be the culprit of cervical cancer [9]. Furthermore, past* in vivo *studies showed that the administration of probiotics are effective in improving lipid profiles, including the reduction of serum/plasma total cholesterol, LDL-cholesterol, and triglycerides or increment of HDL-cholesterol [10]. For example, probiotic* Lactobacillus reuteri* NCIMB 30242 and a few other* Lactobacillus* and* Bifidobacterium *strain have shown that they have potential in reducing serum cholesterol level especially LDL-cholesterol which is established to be one of the major precursors of many chronic diseases including cardiovascular diseases, hypertension, hyperlipidemia, and build-up of atherosclerotic plaque in the arteries [10, 13, 14]. In addition, several other studies demonstrated that probiotic intake reduces the prevalence of allergic diseases including atopic dermatitis rhinoconjunctivitis [11] and asthma [15] as well as alleviating the common symptoms associated with HIV patients [12].

However, the literature on the beneficial impact of probiotic on these diseases is still limited and controversial. In addition, most studies often do not sufficiently address the mechanisms by which probiotics modulate, treat, and reduce the progression of these diseases. Therefore, this review will discuss the association of probiotics in preventing and reducing the prevalence of the aforementioned diseases as well as providing its possible mechanisms of actions.

## 2. Probiotic

Probiotics commonly refer to viable microorganisms which were originated from the gut that has beneficial health impacts on the consumer. Etymologically, the probiotic term appears to be a composite of the Latin preposition* pro *(“for” or “in support”) and the Greek adjective (biotic) from the noun* bios *(“life”) meaning “for life” or “in support of life” [16]. The definition of probiotic has a long evolutionary history. It was first used by Lilley and Stillwell [17] to describe substances secreted by one microorganism that stimulated the growth of another and was later used to describe tissue extracts that stimulated microbial growth and animal feed supplements exerting a beneficial effect on animals by contributing to their intestinal flora balance [18]. Since then, the definition of probiotic had evolved over time and today, probiotic is defined as “live organisms that, when ingested in adequate amounts, exert a health benefit to the host” retaining the previous definitions by Food and Agriculture Organization of the United Nations and World Health Organization (FAO/WHO) with a minor grammatical changes [19]. The revised definition stressed the need for a probiotic to be viable and the experts emphasized that there is no such entity as dead probiotic and if dead organism conveys benefits, it should be referred to a different term. Several hypotheses were elucidated concerning series of the evolvement in probiotics definition. Apart from the advancement on genomic tools that contributes to the identification of new probiotic species and mechanism of actions, involvement of many regulatory organizations and pharmaceutical companies may also contribute to the changes [20].

Most probiotics are commonly known as lactic acid bacteria (LAB) due to their ability to produce lactic acid when fermented with substrate rich in sugar. The LAB was initially subdivided into the genera* Betabacterium, Thermobacterium, Streptobacterium, Streptococcus, Betacoccus, Tetracoccus, *and* Microbacterium* on the basis of their morphologic and phenotypic features. Today, only* Streptococcus *is still retained, whereas most of the others have been renamed into* Lactobacillus, Bifidobacterium*, and* Enterococcus* sp. [21, 22].

*Lactobacillus* refers to Gram-positive rods, lactic acid producing bacteria which are mostly obligate and facultative anaerobes, predominantly found in the human gastrointestinal and genitourinary tracts [23, 24]. On the other hand,* Bifidobacterium *are commonly straight anaerobes, Gram-positive, nonsporing, pleomorphic rod bacteria which produce lactic and acetic acids as the product of carbohydrates fermentation [25, 26]. Compared to* Lactobacillus, Bifidobacterium *is more difficult to be cultivated due to its obligate anaerobes properties and often needs extra maintenance when cultivated in the food product such as yogurt.

Nowadays, the interest in probiotic research and industrialization is on developing consortia of different probiotic species and strain. This is due to the fact that many studies have proven that it delivers superior impact on human health compared to the use of single probiotic strain. For instance, probiotic VSL#3 which contained 8 different mixtures of probiotics was proven to be effective in treating several diseases including ulcerative colitis, irritable bowel disease, diarrhea, improving hepatic insulin resistance in diabetic patients, enhancing the immune system of the consumer, and many more [27–31]. In addition, combinations of* Bifidobacterium infantis* with* Lactobacillus acidophilus* were also proven to be effective in reducing the incidence of necrotizing enterocolitis (NEC) and NEC-associated mortality in critically ill neonates [32]. However, it is very important to ensure that the probiotic consortia do not cross-inhibit among themselves as they will reduce the efficacy of the probiotic product. For instance, a seminal study of a probiotic product containing 15 bacterial showed a significant cross-inhibition of growth among the strains, causing it to be less effective than single strains [33]. Therefore, it is recommended for the probiotic product to be tested in human and any benefits they provide should be stated and supported by peer-reviewed publication in order to ensure the effectiveness of the product [20].

In addition, plenty of bacteria were regarded as a probiotic, but many do not satisfy its desirable properties. According to Mitropoulou et al. [34], several aspects have to be taken into consideration before considering the bacteria as potential probiotic. These aspects include safety and functional and technological characteristics. In order to ensure the safety of the probiotic products, it is essential for the probiotic microorganism to be nonpathogenic and recognized as GRAS for human consumption by US Food and Drug Administration (FDA). These properties are important since some bacteria originated from human GI tract are also pathogenic in nature such as* Helicobacter pylori*,* Clostridium difficile*, and many more.

Furthermore, the functional criteria of probiotics should be established based on both* in vitro *and* in vivo *assays, and the results should be reflected in controlled human studies. The probiotic microbes should also be able to survive in harsh condition of the stomach and GI tract of humans in order to ensure its efficacy [35]. These claims may include the ability of the probiotics to withstand the gastric juice and bile salt. These are due to the fact that many microbes which are claimed as probiotic are not capable of subsisting the acidity level of gastric juices as well as the bile salt. This condition has risen many debates among the probiotic consumer, researcher, and industries. However, several studies have reported that nonviable probiotics could also devour beneficial effects on human health [36, 37]. This is because not all mechanisms nor clinical benefits of probiotic concomitant with viability and even cell wall or the DNA components may have beneficial health impact on human [38]. A study reported that both viable and nonviable* Lactobacillus* bacteria exhibit a similar beneficial effect toward lactose tolerance by lactase-deficient subjects. Similarly, in the treatment of acute gastroenteritis, some probiotics showed clinical efficacy in shortening the duration of diarrhea in both viable and nonviable forms [36].

However, other probiotic species such as* Saccharomyces boulardii *should be in a viable form to show effective effect in candidiasis treatment, differing from most* Lactobacillus* strains that showed efficacy in both viable and nonviable state [36]. Hence, the association of the probiotics viability and its therapeutic impact are still dubious and seem to depend on the microbial species and on the disorder. Therefore, experts suggested that probiotic should best be in a viable form to exhibit a wider therapeutic benefit on human [19]. This is because viable probiotics are able to colonize and adhere at the GI tract of human, providing competitive exclusion of pathogens which therefore maintain the normal intestinal flora. Dead or nonviable probiotic would not be able to provide similar mechanisms and, therefore, their beneficial impact would be limited. In addition, the current definition of probiotic emphasizes the needs of probiotic in a viable form as discussed in the previous sections.

The concern of viability is limited to the ability of the probiotic to withstand not only the bile and gastric juices, but also food production and processing (technological criteria). This is because the viability of bacteria is often reduced during the food manufacture, distribution, and storage. Many surveys have shown large fluctuations and poor viability of probiotic bacteria especially* Bifidobacterium*, in food products, such as yogurt preparations [39, 40]. The sensitivity of* Bifidobacterium* to low pH and hydrogen peroxide as well as with low viability in dairy products during storage remains a major problem in most probiotic products. Therefore, technologies such as immobilization and encapsulation were employed to ensure and maintain the viability and quality of the probiotic product. In general, immobilization and encapsulation of probiotic provide protection of cells against physicochemical changes, such as pH, temperature, bile salts, higher cell densities and cell loads, higher productivity and efficiency, improved substrate utilization, reduced risk for microbial contamination, and faster fermentation and maturation rates [34]. However, these techniques are beyond the focus of this paper; therefore, it would not be discussed in detail.

Apart from safety, functionality, and viability, intake of sufficient probiotic dosage is another key factor to ensure its efficacy on human health. Although the information about the minimum effective concentrations is still limited and controversial, it is generally accepted that probiotic products should have a minimum concentration of 10^6^ CFU/mL or gram and that a total of 10^8^ to 10^9^ probiotic microorganisms should be consumed daily to have an optimal beneficial impact on the consumer [14].

## 3. Association of Probiotic and Its Mechanism on Human Health

To date, the association of probiotic and human health has been well established. The mechanisms underlying the beneficial effects of probiotics on human are largely unknown but are likely to be multifactorial. There are several postulated antagonistic mechanisms of probiotics on pathogenic microorganisms and diseases which may include competing for nutrients as growth substrates, providing and enhancing the gut barrier functions, competitive adherence to the mucosa and epithelium, producing antimicrobial substances, and modulating the immune system [41, 42].

One of the ways probiotics promote human health is by inhibiting the growth of pathogenic bacteria. Probiotics compete for nutrients especially for their growth and proliferation that would otherwise be utilized by pathogens. For example, a sufficient number of probiotics may possibly consume most of the available monosaccharides, which results in the inhibition of pathogenic organism which is solely dependent upon monosaccharide for its growth such as of* Clostridium difficile*. Thus the growth of pathogenic microbes would be stunted and consequently reduce the prevalence of pathogenic bacteria in the GI tract [43]. Furthermore, probiotics enhance the gut barrier function by providing a competitive exclusion for cellular attachment to the mucosa secreted by the epithelial layer of GI the track. Maintaining the epithelial layer is one of a major defence mechanisms of probiotics. This is because once this barrier function is disrupted, pathogenic bacteria and food antigens can extend up to the submucosa and can induce inflammatory responses, which may result in intestinal disorders, such as inflammatory bowel disease [44, 45]. Disruption of intestinal barrier resulted in bacterial translocation which is one of the primary inducers of several types of cancers and other complications. Several studies demonstrated that probiotics such as* Lactobacillus rhamnosus *strain GG and* Lactobacillus plantarum *299 showed the ability to inhibit attachment of enteropathogenic* Escherichia coli *in the GI tract [43, 46]. In addition, a number of* Lactobacillus *bacteria modulate and enhance the expression of genes involved in tight junction signalling, such as E-cadherin and *β*-catenin, to reinforce the intestinal barrier integrity. Probiotics do maintain the intestinal barrier integrity by anchoring and adhering to the intestinal mucosa. Several* Lactobacillus *proteins have been shown to promote mucous adhesion by displaying surface adhesins and integrate with complex glycoprotein mixture (i.e., mucin) secreted by the intestinal epithelial cell to provide competitive exclusion of pathogens from the mucus [41].

Another proposed mechanism of probiotic is the modification of the microbial flora through the synthesis of low molecular weight compounds such as organic acid as well as large molecular weight antimicrobial compounds termed as bacteriocins [41]. Examples of organic acids are acetic and lactic acids. These compounds have been proven to exhibit strong inhibitory effect against pathogenic Gram-negative bacteria such as* Helicobacter pylori* which has been implicated with many gastrointestinal disorders. The mode of actions of these acids includes lowering the intracellular pH or accumulating the ionized form of the organic acid which will disrupt the pH balance of the pathogen and consequently inhibit the growth of the pathogen [47, 48]. Furthermore, probiotics also produce bacteriocins and other compounds. Bacteriocins are compounds produced by bacteria that have a biologically active protein moiety and antibactericidal activity. The example of bacteriocins produced by probiotics are lactacin B from* L. acidophilus*, bifidocin B produced by* Bifidobacterium bifidum *NCFB 1454, plantaricin from* L. plantarum*, and nisin from* Lactococcus lactis *[49]. These compounds were proven to be effective against food-borne pathogen and its common mechanism includes destruction of target cells by pore formation and/or inhibition of cell wall synthesis. For instance, bifidocin B, which is produced by* B. bifidum *NCFB 1454, exerts a strong inhibitory activity against several pathogenic bacteria, including* Salmonella enterica *ser.* typhimurium *SL1344 and* E. coli *C1845 [50].

It is well known that probiotic bacteria can stimulate the immune response by modulating the adaptive and innate responses of the host [41]. The adaptive immune response depends on B and T lymphocytes, which are specific for particular antigens whereas innate immune system responds to common structures called pathogen-associated molecular patterns (PAMPs) shared by the vast majority of pathogens. The primary response to pathogens is triggered by pattern recognition receptors (PPRs), which bind PAMPs. The best-studied PPRs are Toll-like receptors (TLRs). TLRs are transmembrane proteins expressed on various immune and nonimmune cells, such as B cells, natural killer cells, dendritic cells (DC), macrophages, fibroblasts, epithelial cells, and endothelial cells. It is well established that probiotics can suppress intestinal inflammation via the downregulation of TLR expression, secretion of metabolites that may inhibit TNF-*α* from entering blood mononuclear cells, and inhibition of NF- *κ*B signalling in enterocytes [51]. In a study,* Lactobacillus casei* ATCC27139 has significantly enhanced the innate immune response of mice by phosphorylation of NF-*κ*B, p65, p3, MAPK, and MAPKAPK-2 signalling pathway [52]. In addition, another study found that probiotic mixture VSL#3 elicited noninflammatory responses from epithelial and immune cells, inhibited IL-8 and systemic TNF- *α* production, and improved the histological score of inflammation in IL-10 knockout mice [53]. Furthermore, probiotics can encounter DCs, which have an important role in innate and adaptive immunity. Both intestinal epithelial cells (IECs) and DCs can interact with and respond to gut microorganisms through their PPRs [41].

However, most preventive and therapeutic mechanism of probiotics are generally species and diseases specific. Therefore, in this review, we are going to discuss the potential role of probiotics in reducing the prevalence of cancer, hypercholesterolemia, dermatitis, and allergic symptoms, and common symptoms associated with HIV-infected individual and providing their possible mechanisms of actions.

## 4. Probiotic and Cancer

In 2012, cancer is classified as the second major death cause in different regions of the world with an estimated number of 14.1 million new cases and 8.2 million death and expected to increase up to 21 million cases with 13.2 million causalities by 2030. Cancer is caused by a progressive aggregation of mutations in the genetic material of cell. Uncontrolled proliferation of cells, insensibility of growth factors, and capacity to infect surrounding tissues are the general characteristic of malignant tumors observed in most cancer patient [69–72]. According to Anand et al. [73], only 5-10% of all cancer cases can be attributed to genetic defects, while 90-95% of the cases are related to external factors. According to the World Cancer Report (2014), around one-third of all deaths caused by cancer are resulting from high body mass, low fruits and vegetable intake, sedentary lifestyle, tobacco intake, and alcohol ingestion [74].

The association of probiotic in preventing, treating, and reducing the progression of cancer cell has been established years ago. Extensive research using human cancer cells/cell lines has proven that probiotics possess antiproliferative or proapoptotic activities in on a wide range of cancer cells including colon, stomach, breast, cervix, and myeloid leukaemia cells [7, 75–85]. According to Russo et al. [76] and Orlando et al. [77], probiotic* Lactobacillus rhamnosus* strain GG (LGG) and* Bifidobacterium adolescentis* SPM0212 showed a significant antiproliferative role and inhibit human gastric cancer cells and three colonic cancer cells lines including HT-29, SW 480, and Caco-2. Another study [85] found that the kefir product containing* Lactobacillus kefiri *possessed apoptotic effect on myeloid leukaemia cell lines. In addition,* Enterococcus lactis *IW5 which was obtained from human gut strongly inhibited the growth of several pathogenic bacteria and decreased the viability of different cancer cells, such as HeLa, MCF-7, AGS, HT-29, and Caco2, representing the potential therapeutic effect of probiotic on cancer patients [86].

The anticancer effect of probiotic on cancer patients was demonstrated in Table 1. Probiotic treatments have been shown to be effective in preventing, treating, and reducing the progression of several types of cancers including colorectal, liver, breast, bladder, colon, and cervical in cancer patients (Table 1). Due to these reasons, probiotics-based regimens are often used as an adjuvant during anticancer chemotherapy treatments.

However, the potential mechanisms of probiotic in preventing, treating, and reducing the progression of cancer are still poorly understood and need to be further elucidated. Up to date, there are several reported and established mechanisms in cancer prevention and treatment by probiotic which may include (i) modulation of gut microbiota, (ii) enhancement of gut barrier functions, (iii) degradation of potential carcinogens and protection effect of DNA damage of intestinal epithelium, and (iv) enhancement of immune and inflammatory system in the body.

*(i) Microbiota Modulation. *One of the potential mechanisms of probiotic is modulating the composition of gut microbial species by maintaining the balance and suppressing the growth of potential pathogenic or cancer-inducing bacteria in the gut. For example, several Gram-positive probiotic can synthesize antimicrobial peptides, acetic, lactic, and propionic acid which reduce the intestinal pH and consequently inhibit the growth of several pathogenic Gram-negative bacteria [87]. These data were further supported by several other studies [88–90] which showed that several strains of lactobacilli have antagonistic activities against Gram-negative gastric-cancer-related* Helicobacter pylori*. Furthermore, another study found that some* Lactobacillus *strains produce lactic acid which inhibits the growth of* Salmonella enterica *[91]. In the Simulator of the Human Intestinal Microbial Ecosystem (SHIME) model,* L. acidophilus* or* L. casei* increased LAB with a concomitant decrease of fecal coliforms and clostridia [92]. In addition, a study reported by Li et al. [93] found that probiotics caused shifts in the gut microbiota composition toward specific beneficial bacteria, for example,* Prevotella *and* Oscillibacter*. These bacteria are known to produce anti-inflammatory metabolites, which subsequently decreased the Th17 polarization and favoured the differentiation of anti-inflammatory Treg/Type 1 regulatory T (Tr1) cells in the gut.

*(ii) Enhancement of Gut Barrier Function. *Maintaining the gut epithelial barriers is crucial as it maintains a peaceful relationship with commensal microorganisms while protecting the host from pathogens and pathobionts. Dysbiosis, which is an alteration in the composition of the gut microbiota associated with pathology, disrupts the physiological interaction between epithelial cells and the microbiota, results in breaching of the barriers, inducing inflammatory pathologies, and may contribute to cancer initiation and progression [94]. Commane et al. [95] indicated that the fermentation products of pro- and prebiotics prevented disruption of the intestinal epithelial barrier, while Ko et al. [96] demonstrated that* L. plantarum* inhibited the decrease in transepithelial resistance of Caco-2 cells. Administration of probiotic products to patients undergoing biliary drainage reduces the intestinal permeability and attenuated the inflammatory response [97, 98]. Probiotic has also proven to enhance the expression of tight junctions' protein such as mucin gene (MUC2 and MUC3), which will enforce and enhance the intestinal gut barrier functions [41]. These data suggest a protective role of probiotic role in maintaining the mucus layer integrity, which is essential for an effective intestinal barrier function.

*(iii) Degradation of Potential Carcinogens and Protective Effect of DNA Damage. *Carcinogens such as 2-dimethylhydrazine (DMH) and N-nitrosodimethylamine (NDMA) are substances which can cause changes in the DNA sequence which may lead to tumorigenesis. The potential of probiotic in degrading carcinogenic compound has been studied substantially [99]. A study on the effect of freeze-dried probiotics supplementation which consists of* Lactobacillus acidophilus* Delvo Pro LA-1,* Lactobacillus rhamnosus* GG, B*ifidobacterium animalis *CSCC1941, and* Streptococcus thermophilus* DD145 strains on 100 DMH-induced intestinal tumors rats showed significant inhibition of tumors within the rat colon compared to control group [100].

In addition, probiotic is also proven to decrease mutagen-induced DNA damage or DNA adduct formation in the colonic epithelium [101–104]. An in vitro study using rat intestinal epithelial cells showed preventive role of probiotics against enterocyte apoptosis and loss of intestinal barrier function caused by 5-fluorouracil (5-FU) [105], while an in vivo study with rats demonstrated that combination of resistant starch and* B. lactis* facilitated the apoptotic response to carcinogen-induced DNA damage of the rat colorectal cells [106]. The administration of probiotics or synbiotics significantly decreased the activities of intestinal procarcinogen enzymes which was associated with colonic carcinogenesis in experimental animal models [107–109]. Administration of a probiotic bacteria,* Bacillus polyfermenticus*, significantly reduced the number of DMH-induced ACF in F344 rats, when compared to the controls (DMH-treated, no probiotics supplementation) [110]. Furthermore, a study conducted by Ohkawara et al. [111] reported that the probiotics-treated group showed significantly less DMH-induced DNA damage, less blood lipid peroxidation, and increased Total Radical Trapping Antioxidant Potential (TRAP) by 9.3 % versus the controls.

*(iv) Enhancement of Immune System, Signalling Pathway, and Reduction of Inflammatory Reaction. *Series of studies have proved that probiotic enhances the immune system of a cancer patient. Lakritz et al. [112] reported that* Lactobacillus reuteri* ATCC-PTA-6475 inhibited mammary carcinogenesis in wild-type and FVB strain erbB2 (HER2) (genetically susceptible to mammary tumors mimicking breast cancer in human) mutant mice by triggering CD4+ and CD25+ lymphocytes. Another study reported that supplementation of* Lactobacillus casei *strain Shirota enhance NK and T cells activities and improve the phagocytic activity of macrophages which consequently inhibit cancer progression in mice infected with a various type of cancers [113–115]. Study on patients with colon cancer showed that oral administration of* Bacillus polyfermenticus* stimulates IgG production and modulates the number of CD4þ, CD8þ or NK cells [7].

Other studies has also speculated that incorporation of probiotics in the diet has a substantial impact on cell signalling system of the cancer patients.* L. reuteri* may prevent carcinogenesis via downregulating NF-*κ*B-dependent genes which regulate cell proliferation (Cox-2, cyclin D1) and survival (Bcl-2, Bcl-xL) [116]. In another study, 150 patients with colorectal carcinoma administered with probiotic showed significant reduction of the disease complication through inhibition of p38 mitogen-activated protein kinase signalling pathway compared to control group [54]. In addition, a novel purified* Lactobacillus acidophilus* 20079 exopolysaccharide, LA-EPS-20079 inhibit in human colon cancer by molecularly regulates both apoptotic and NF-*κ*B inflammatory pathways [117].

Furthermore, inflammation causes cancer development through processes that involve genotoxicity, aberrant tissue repair, proliferative responses, invasion, and metastasis. Major inflammatory pathways that are involved in inflammation-induced carcinogenesis converge at the level of the transcription factors signal transducer and activator of transcription 3 (STAT3) and nuclear factor-*κ*B (NF-*κ*B) [118]. Probiotics have been shown to have anti-inflammatory activities through regulating the production of inflammatory mediators such as interleukins, interferons, and cytokines resulting in the effective control of inflammation and carcinogenesis [106]. For instance, Matsumoto et al. [119] and Appleyard et al. [120] have shown that supplementation of* Lactobacillus* and VSL#3 probiotic reduce and delay transition from inflammation to dysplasia in a rat model of colitis-associated cancer.

## 5. Probiotic and Cholesterol

The other popular application of probiotic in maintaining human health is by reducing serum cholesterol level in blood. High content of low-density lipoprotein cholesterol (LDL-C) is a major precursor to hypertension, hyperlipidemia, and coronary heart diseases as well as causing build-up of atherosclerotic plaque in the arteries [9]. Therefore, by maintaining the serum LDL-cholesterol level at the optimal ranges, the chances of getting the aforementioned diseases may possibly be reduced significantly.  Table 2 showed several randomized controlled clinical trials on the hypocholesterolemic effect of probiotics on human subjects [58–65]. In another elegant study, pooled data from 485 participants which were divided into 3 groups; “high”, borderline high, and normal serum cholesterol levels in randomized controlled clinical trials suggested that probiotic consumption significantly lowered LDL-C and total cholesterol levels among all categories, compared to the control group [121]. This study further supported the beneficial impact of probiotic consumption in reducing serum cholesterol level in human.

However, most studies on the efficacy of probiotic in modulating cholesterol level often do not sufficiently address its mechanisms. Therefore, hypocholesterolemic mechanism of probiotic will be discussed in this review. An elegant review by Ooi and Liong [122] demonstrated several cholesterol-lowering mechanisms of probiotics which are summarized in this review. The mechanisms include the following.

*(i) Enzymatic Deconjugation of Bile Acids by Bile Salt Hydrolase. *Bile consists of cholesterol, phospholipids, conjugated bile acids, bile pigments, and electrolytes. Probiotics reduce the cholesterol level by deconjugating the bile salt. Deconjugation of the bile salt causes it to be less soluble and absorbed by the intestines, leading to their elimination in the faeces. Cholesterol is then used to synthesize new bile acids in homeostatic response, resulting in lower serum cholesterol in the blood [123].

*(ii) Ability to Bind Cholesterol in the Small Intestines. *The ability of cholesterol-binding of probiotic appeared to be growth and strain specific. Hosono [124] reported that several probiotic such as* Lactobacillus gasseri* has the ability to remove cholesterol from a laboratory media via binding onto its cellular surfaces. Furthermore, Kimoto et al. [125] have further strengthened the finding by showing that both living and dead probiotics have the ability to reduce the cholesterol level by exerting the similar mechanism. However, the authors found that growing cells removed more cholesterol than dead cells.

*(iii) Assimilation and Incorporation of Cholesterol into the Cellular Membranes of Probiotics. *A study [125] found that several probiotic* lactococci* strains reduce cholesterol level by assimilating and incorporating the cholesterol into their cellular membranes during the growth phase, thus lowering cholesterol level in blood. Incorporation of cholesterol into the cellular membrane benefits the bacterial strain by increasing its membrane strength and subsequently lead to higher cellular resistance toward lysis.

*(iv) Converting Cholesterol into Coprostanol. *Furthermore, probiotic could also reduce the cholesterol level by converting it into coprostanol. The coprostanol will then directly excrete in faeces, resulting in decreases amount of cholesterol being assimilated from the body. The probiotic possibly initiated the conversion of cholesterol into coprostanol by producing cholesterol dehydrogenase/isomerase which transforms cholesterol to cholest-4-en-3-one, an intermediate cofactor in the conversion of cholesterol to coprostanol [126].

*(v) Decrease the Concentration of Cholesteryl Esters in LDL Particles. *Administration of a synbiotic containing* L. acidophilus *ATCC 4962, fructooligosaccharides, inulin, and mannitol in hypercholesterolemic pig reduced total cholesterol by lowering concentration of cholesteryl esters in the LDL particles and a higher concentration in triacylglycerol [127]. Triacylglycerol-enriched LDL particles are more susceptible to hydrolysis and removal from blood, while the loss of cholesteryl esters forms smaller and denser LDL particles leading to higher removal rates from blood compared to larger LDL particles.

By reducing cholesterol level in the blood, the risk of developing coronary heart disease, hypertension, atherosclerosis, heart attack, and stroke is reduced by nearly half. Therefore, probiotic supplementation could be a potential adjuvant for coronary heart disease, hypertension, atherosclerosis, heart attack, and stroke treatment.

## 6. Probiotic and Allergy

An allergy occurs when a person's immune system reacts with the substance in the environment known as an allergen that is harmless for most people. Allergic reactions may include anaphylaxis, asthma, and contact dermatitis as well as being allergic to drug, food, latex, seasonal, mold, and animals. Although most types of allergic reactions are not medically severe, some of them such as asthma, atopic dermatitis, anaphylaxis, and rhino conjunctivitis (atopic diseases) may be life-threatening without a proper treatment [128]. Recent evidence suggests that exposure to bacteria in early life may exhibit a protective role against allergy. Several studies demonstrated that the guts of children who were born through vaginal and breastfed are prone to be colonized with* Bifidobacteria *and* Lactobacillus* bacteria while the caesarean and formula-fed infant, however, tend to have a flora that is more complex, consisting mostly of* Coliforms* and* Bacteroides*. Therefore, most caesarean and formula-fed infant have a significantly lower prevalence of* Bifidobacteria *and they are normally associated with frequent respiratory allergies [129]. This study indicated that the composition of gut bacteria has a significant role in allergy prevention and treatment. Therefore, there has been obvious interest in the potential benefits of modifying the gastrointestinal flora by using probiotic supplementation for allergy treatment.

Until now, several randomized, double-blind, placebo-controlled studies found that supplementation of probiotics have a significant influence in treating patients with allergy or atopic diseases including eczema, rhinoconjunctivitis, atopic dermatitis, allergic rhinitis, and asthma (Table 3). However, not all probiotic bacteria are effective in preventing/treating allergic reaction and most of the mechanisms of action are species and strain specific as well as time-dependent. For instance, supplementation of* Bifidobacterium animalis subsp lactis *HN019 with the dose of 10^9^ CFU/day for 4 years had no effect on infant with eczema disease whereas supplementation of* Lactobacillus rhamnosus *HN001 with the similar study design has shown significant protective effect and reduced the prevalence of eczema and rhinoconjunctivitis by 50% compared to control [66]. This study suggested the importance of choosing the right probiotic species and strain to ensure its efficiency for allergic treatment.

**Figure 1:**
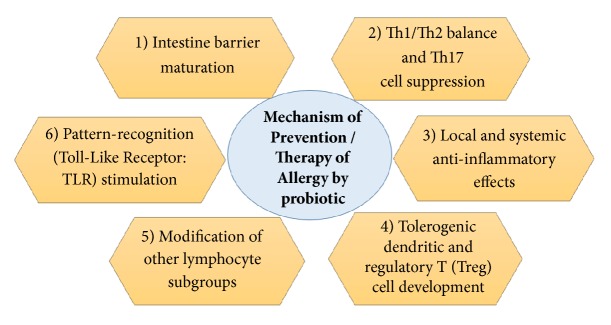
Potential antiallergic mechanism of probiotic on host.

Even though the favourable effects of probiotics on various allergic and atopic diseases have been considered for decades, little is known about how probiotics modify the immune system and atopic disease development. Recently, Özdemir [130] has described the potential mechanism of probiotics in preventing and treating allergy as shown in Figure 1. The possible mechanism includes intestinal barrier maturation and immune response modulation by balancing Th1/Th2 ratio while suppressing Th17 cells, local and systemic anti-inflammatory effects, Tolerogenic Dendritic and Regulatory T (Treg) cell development, and modification of other lymphocyte subgroups, as well as pattern recognition receptor (TLR) stimulation [130].

Probiotics induce intestinal barrier maturation by providing the maturational signal for the gut-associated lymphoid tissues (GALT). Furthermore, some probiotics are able to alter the cytokine profiles released by peripheral blood mononuclear cells and redirect the immune system in a regulatory or tolerant mode which will provide balance in Th1/Th2 productions as well as suppressing the proinflammatory cytokines (Th17 cells) productions. Probiotic also provides local and systemic anti-inflammatory impact by increasing the secretions of IL-10 in the gut and reducing the productions of proinflammatory cytokines, e.g., IFN-*γ*, TNF-*α*, and IL-12 [130].

In addition, recent research suggested that probiotics are involved in the development of Tolerogenic dendritic and regulatory T (Tregs) cell differentiation which will reduce the prevalence of the allergic reactions. Moreover, T cells induced by* Bifidobacterium bifidum* may drive dendritic cells as generators of more IL-10 [27]. Probiotics do exhibit their actions by modifying other lymphocyte subgroups. A study showed that probiotic consumption reduces CD4+ and the CD25+ cells counts and amplified CD8+ cells which improve the prevalence of eczema in preschool children. Finally, probiotic consumption stimulates Toll-like receptor activity. The TLR-mediated actions of probiotics require immunoregulatory cytokines, e.g., IL-10 and TGF-*β* and diverse subgroups of Treg cells, particularly CD4+, CD25+, FoxP3+ cells for TLR-4 stimulators, and NKT cells for the TLR-3 stimulator [130].

## 7. Probiotics and HIV

HIV-infected individuals often have impaired gastrointestinal (GI) tract which leads to microbial translocation and consequently contributes to chronic immune activation and disease progression. Although antiretroviral (ARV) treatment of HIV-infected individuals improves their prognosis, ARV-treated individuals still have increased morbidity and mortality compared with uninfected individuals [131]. Supplementation of probiotics has shown to be beneficial in treating and preventing GI-associated diseases such as diarrhea, irritable bowel diseases, and much more by enhancing the gut barrier function, thus preventing bacterial translocation from the gut [41]. Therefore, since HIV-infected individuals have significantly impaired GI tract, the potential of probiotics in improving and slowing the progression of the diseases would be discussed in this review. Apart from bacterial translocations, the other hallmark in HIV-infected individual includes severe diarrhea, a significant decrease in CD4 cells, and bacterial vaginosis [132] which would also be deliberated in this review.

A study has been conducted to elucidate the impact of probiotic consumption on bacterial translocation among HIV-infected individuals [133]. The study was performed with 44 HIV+ patients who was administrated with* S. boulardii* over 4 weeks, the results showed that LPS-binding protein (a marker of translocation) and IL-6 (a marker of inflammation) were significantly decreased compared with placebo recipients. Another study which was carried out toward HIV-infected individuals in Mwanza, Tanzania, established that yogurt supplemented with* Lactobacillus rhamnosus* effectively alleviates GI symptoms and improves productivity, nutritional intake, and tolerance to antiretroviral treatment among the patients [134]. However, a meta-analysis study by Carter et al. [132] determined that the effect of probiotic on bacterial translocation toward HIV patients was inconclusive and suggested that further studies should be contemplated.

The other symptom of individual with impaired GI tract is severe diarrhea and it is commonly associated with the HIV-infected subject. HIV-infected patient supplemented with probiotic in their diet have shown significant improvement and alleviation of diarrhea. A study conducted by Anukam et al. [135] found that 18 out of 24 HIV^+^ adult women who experience severe diarrhea with flatulence and nausea significantly alleviate the symptoms within 30 days after being supplemented with probiotics. However, the symptoms reappear after 3 months of discontinuing the treatment, indicating the importance of continuous consumption of probiotics to experience persistent beneficial impact. In another pilot study conducted for 12 weeks with 35 HIV^+^ adults with diarrhea showed that 36% of the patients resolved the diarrhea symptom completely after being supplemented with probiotic and glutamine in their diet [136]. Furthermore, another study conducted with 171 HIV+ adults with approximately 60% of them on ARV who consume yogurt for 3 years have experienced significantly less ARV- related stomach pain as well as fewer GI symptoms that affects daily life [137]. Probiotics also have shown to benefit and treat the symptom in HIV+ children. A meta-analysis study in children infected with HIV disease shows significant decrease in the duration of diarrhea and fever [138]. These studies concluded that probiotics significantly improve the diarrhea symptom in adults and children infected with HIV.

The other hallmark of patients infected with HIV diseases is that they have a significantly low number of CD4 cells count. This is due to the fact that HIV virus uses CD4 cell to reproduce and proliferate which leads to significant decrease in CD4 counts and consequently damage the immune system of the infected individuals. Supplementation with probiotics has shown to improve the CD4 counts in HIV-infected individuals. A study found that 8 out of 12 ARV-naive patients who consumed probiotic yogurt daily for 15 days increase the CD4 counts up to 4-fold compared to placebo group [135]. A meta-analysis review by Carter et al. [132] found that 4 out of 7 studies on HIV+ patients who received ARV treatment show profound increase of 62 CD4/year after including probiotic in their daily regime. In addition, another study reported that women with CD4 <200 showed a significant increase of CD4 cells with the mean of 93 cells/*μ*L versus a mean decrease of 69 cells/*μ*L among placebo recipients [139]. However, 2-week and 3-month study of 17 patients showed no impact on their CD4 cell counts, proposing that probiotics only improve CD4 counts modestly among HIV-infected individuals.

Finally, the impact on probiotic supplementation among HIV-infected women with bacterial vaginosis was also elucidated in this review. Bacterial vaginosis is a condition in which vaginal flora which is commonly dominated by* lactobacilli *gradually shifted to a complex mix of pathogenic anaerobic bacteria such as* Gardnerella vaginalis* and* Mycoplasma hominis*. This condition has been reported to facilitate disease progression as well as the transmission of HIV disease [140]. A randomized, double-blind, placebo-controlled study conducted with 65 HIV-infected women (Nugent score between 4 to 10) supplemented with* Lactobacillus rhamnosus *GR1 and* Lactobacillus reuteri* RC daily or placebo for 6 months were elucidated. The author found that these probiotics did not enhance the cure of this diseases among women with HIV but may prevent disease progression among the populations. Therefore, probiotics may be used as an adjuvant for bacterial vaginosis treatment for HIV patients in order to prevent it from spreading [141].

In conclusion, supplementation of probiotic has been shown to be beneficial in alleviating the common symptoms associated with HIV patients. Given the paucity of evidence for adverse events, low cost, and potential for economic value to people living in poverty [138], the use of probiotics in treating and preventing further progression of HIV-infected individual seems to be practical and feasible. Restoration of gut flora to a more healthful ecology may have several important clinical benefits particularly in conjunction with improved nutrition and access to micronutrient supplementation which will result in a better health among the HIV-infected individuals.

## 8. Conclusions

Probiotics have obtained increasing medical importance because of their beneficial effects on the host health. Oral administration of probiotics has multiple effects such as normalization of the intestinal microflora, improvement of the gastrointestinal barrier, and inhibition of potential pathogens or carcinogenesis in the gut. Together with the enhancement of systemic immune or/and anti-inflammatory activities, probiotics may play a part in reducing the risk of multiple chronic diseases including cancer, high serum cholesterol-associated diseases, and allergic and HIV diseases. Nevertheless, beneficial effects of probiotic on the aforementioned diseases on human are still controversial due to several factors including inappropriate design of study, inadequate concentration of effective dosage, interaction with nutritional components in food, and the utilization of animal models without further confirmation with human clinical trials. Also, the use of animal models does not always adequately reflect what occurs in the human body, since their metabolism may be significantly different. Therefore, it is recommended that further studies, especially long-term human complementary studies, should be addressed to clarify the contention.

## Figures and Tables

**Table 1:** Anticancer effect of probiotic on cancer patients.

**Probiotic strain**	**Subjects**	**Dose and duration of study **	**Effects (P<0.05)**	**Ref**
*Lactobacillus plantarum *CGMCC, *Lactobacillus acidophilus*-11 *and Bifidobacterium longum*-88	(i) Colorectal cancer patients (ii) 150 patients (1;1 ratio of probiotic and placebo group)	(i) *Lactobacillus plantarum* CGMCC no.1258;10^11^(CFU)/g (ii) *Lactobacillus acidophilus;*10^11^CFU/g, (iii) *Bifidobacterium longum-*8810^10^ (CFU/g) (iv) The patients administrated with probiotic 6 days preoperatively and 10 days postoperative.	Probiotics decreased the serum zonulin concentration, duration of postoperative pyrexia, duration of antibiotic therapy, and rate of postoperative infectious complications as well as inhibited the p38 mitogen-activated protein kinase signalling pathway.	[54]

*Lactobacillus rhamnosus* LC705 and *Propionibacterium freudenreichii* *subsp. shermanii strains*	(i) Aflatoxin-induced liver cancer (ii) 90 male students with high aflatoxin level in urine	5 weeks, (1:1, wt: wt) at a dose of 2–5×10^10^ colony-forming units/day	61.5% reduction of a liver cancer biomarker which leads to reduced urinary excretion of aflatoxin B1-N7guanine (AFB-N7-guanine)	[55]

*Lactobacillus casei Shirota*(LcS)	(i) Breast cancer (ii) 968 breast cancer patients (306 probiotic group; 662 control) aged 40 to 55.	Frequent consumption of Yakult containing *Lactobacillus casei Shirota* and isoflavones from soy product for 2 years	Regular consumption of LcS and isoflavones since adolescence was inversely associated with the incidence of breast cancer in Japanese women.	[56]

*Lactobacillus acidophilus *L1	(i) Bladder cancer (ii) A total of 180 cases (mean age: 67 years, SD 10) and 445 population-based controls	200 g of yoghurt containing *L. acidophilus *L1 for 10 weeks	Habitual intake of lactic acid bacteria reduces the risk of bladder cancer.	[57]

*Lactobacillus casei Shirota* (LcS)	(i) Cervical cancer (ii) 54 women with an HPV-positive intra epithelial lesion	Daily administration of (Yakult) containing LcS for 6 months.	60 % reduction in human papilloma virus (HPV) associated infection and cervical cancer precursors.	[8]

**Table 2:** Hypocholesterolemic effects of probiotic on human.

**Probiotic strain**	**Subjects**	**Dose and duration of study **	**Effects (P<0.05)**	**Ref.**
*Lactobacillus plantarum ECGC 13110402*	49 normal to mildly hypercholesterolaemic adults	2x10^9^ CFU encapsulated *Lactobacillus plantarum *ECGC 13110402 twice daily for 16 weeks.	(i) Significant reduction in LDL-C in volunteers with baseline TC<5mM during the 0±12 week period (13.9%) (ii) Significant reduction in TC in volunteers with baseline TC_6mM in the 0±6 week period (37.6%) (iii) A significant decrease in TAG (53.9%) (iv) increase in HDL-C (14.7%) in the over 60 years population in the 6±12 week period	[58]

*Lactobacillus fermentum ME-3*	45 clinically asymptomatic hypercholesterolaemic participants	Consumed an RAC containing an anti-oxidative and anti-atherogenic probiotic *Lactobacillus fermentum* ME-3 (LFME-3) for 4 weeks.	(i) The reduction of total cholesterol (from 6.5 ± 1.0 to 5.7 ± 0.9 mmol/l) (ii) HDL cholesterol level rose from 1.60 ± 0.31to 1.67 ± 0.34mml/l)	[59]

*Bifidobacterium lactis *HN019	51 subjects with metabolic syndrome (MetS) other cardiovascular risks	Daily ingestion of 80 mL fermented milk with 2.72 x 10^10^ CFU of *B. lactis *HN019 for 45 days.	(i) 7.7 % decrease of total cholesterol (ii) LDL-cholesterol reduce by 13%	[60]

*Streptococcus thermophilus*	30 subjects, respectively, with average serum LDL-cholesterol levels of about 140 mg/dl.	*Streptococcus thermophiles *(ST) -fermented milk or non-fermented placebo milk (PC) was consumed once a day for 12 weeks	Daily consumption of ST-fermented milk beneficial in healthy or mildly hyper- LDL cholesterolaemic subjects. The benefits were particularly remarkable in subjects who had higher levels of MDA-LDL.	[61]

*Lactobacillus acidophilus *L1	48-volunteers with serum cholesterol level ranging from 5.40 to 8.32 mmol/L	200 g of yoghurt containing *L.* *acidophilus *L1 for 10 weeks	Reduction of 2.4% total cholesterol level compared to the placebo group.	[62]

*Bifidobacterium longum*BL1	32 subjects with baseline cholesterol ranging from 220-280 mg/dL, body weight ranging from 55.4-81.8 kg, aged 28-60 years old	Incorporation of 10^8^ CFU/g *B. longum*BL1 daily for 4 weeks.	(i) a significant decline in serum total cholesterol, LDL-cholesterol and triglycerides (ii) 14.5% increase in HDL-cholesterol	[63]

*Lactobacillus plantarum* 299v (ProViva)	36 healthy volunteers with moderately elevated fibrinogen concentrations (>3.0 g/L); 35-45 years old; mean total cholesterol of 5.59 ± 0.88 mmol/L for treatment group & 5.51±0.75 mmol/L for control group.	400 mL of rose-hip drink containing 5.0 × 10^7^ CFU/mL daily for 6 weeks.	(i) 2.5% decrease in total serum cholesterol level (ii) Decrease in LDL-C by 7.9%	[64]

*Enterococcus faecium*& 2 strains of *Streptococcus* *thermophilus* (Causido®; Gaio®)	32 patients between 36-65 years old with mean total cholesterol of 48.47 ± 26.75 mg/dL and mean LDL-C of 172.22±21.17 mg/dL	200 g of Gaio® containing 10^5^-10^9^ /mL of *E. faecium*& 5-20× 10^8^/mL of *S. thermophilus* daily for 16 weeks	(i) Total cholesterol reduced by 5.3% (ii)LDL cholesterol reduce by 6.15%	[65]

**Table 3:** Potential antiallergic effect of probiotic on human.

**Types of allergy**	**Probiotic microorganisms **	**Outcomes**	**Ref.**
Eczema and rhinoconjunctivitis	*Lactobacillus rhamnosus HN001*	Supplementation of *Lactobacillus rhamnosus* to high-risk infant for 4 years significantly shows protective effect and reduce the prevalence of eczema and rhinoconjunctivitis by 50%.	[66]

Atopic dermatitis	*Lactobacillus plantarum *CJLP133	Fourteen weeks supplementation of *L. plantarum* CJLP133 showed beneficial impact to all the atopic dermatitis patients compared to the control group.	[67]

Allergic rhinitis	*Lactobacillus salivarius*	The study found that *Lactobacillus salivarius* treatment reduces rhinitis symptoms and drug usage in children with allergic rhinitis.	[68]

Asthma and allergic rhinitis	*Lactobacillus gasseri*	Pulmonary function and peak expiratory flow rates (PEFR) increased significantly, and the clinical symptom scores for asthma and allergic rhinitisdecreased in the probiotic-treated patients as compared to the controls.	[12]
